# Impact of endodontic irrigants on surface roughness of various nickel-titanium rotary endodontic instruments

**DOI:** 10.1186/s12903-023-03227-0

**Published:** 2023-07-24

**Authors:** Tamer M. Hamdy, Yasmine Mohsen Alkabani, Amira Galal Ismail, Manar M. Galal

**Affiliations:** Restorative and Dental Materials Department, Oral and Dental Research Institute, National Research Centre (NRC), Giza, Dokki, 12622 Egypt

**Keywords:** Atomic Force Microscopy, Surface roughness, Irrigation, Sodium Hypochlorite, Ni-Ti, rotary instruments

## Abstract

**Background:**

The aim of the current study is to assess the surface roughness of several recent nickel-titanium (Ni-Ti) rotary endodontic instruments, namely: Protaper next (PTN); Hyflex CM (CM); Hyflex EDM (EDM); WaveOne gold (WOG); and trunatomy (TN), before and after application of 5.25% sodium hypochlorite (NaOCl) irrigant solution.

**Methods:**

In this in vitro study, five recently introduced rotary endodontic instruments of different metallurgical properties and designs were subjected to Atomic Force Microscopy (AFM) analysis, and then each file was rotated in 5.25% NaOCl for 15 min., with speed and torque according to manufacturer’s instructions. The instruments were then subjected to AFM analysis again. The surface roughness average (Sa) parameter was calculated. Data were analyzed by Paired T test, One-way ANOVA and Tukey tests.

**Results:**

There was a statistically significant decrease in the surface roughness of all rotary endodontic instruments after immersion in irrigants (P ≤ 0.05).

**Conclusion:**

The new TN and PTN instruments showed the least surface roughness. All tested Ni-Ti rotary endodontic instruments after irrigants exposure showed a varying increase in surface roughness.

## Background

Several manufacturing approaches and modifications have been developed to enhance mechanical properties and reduce the incidence of rotary file fracture, which led to effective endodontic treatment outcomes [1–3]. The process of Ni-Ti alloy cold working induces stressed areas that are predisposed to the formation of cracks or brittle fractures [4, 5]. Therefore, continuous improvements in Ni-Ti endodontic instruments could be achieved through thermal, mechanical, and electrical treatment to impart a more stable martensite phase into the Ni-Ti endodontic instruments at normal mouth temperature, allowing maximum flexibility to be gained [6]. Moreover, heat treatment could also improve their resistance to cyclic fatigue compared to conventional Ni-Ti alloys. Ni-Ti endodontic instruments could be described as those mostly comprising the austenitic phase and those mostly containing the martensitic phase [7]. Endodontic instruments based on austenitic alloys possess superelastic properties. On the contrary, endodontic instruments based on martensitic alloys (M-wire) demonstrated a shape memory effect, became ductile, easily deformed, and exhibited more flexibility [6, 7].

The Ni-Ti instrument’s surface topography could be altered by manufacturing composition, heat treatment, geometrical designs, and mechanical stresses [2, 5]. For example, HyFlex CM (CM) rotary instrument (HCM; Coltene/Whaledent AG, Altstätten, Switzerland) is a controlled shape memory wire, has a stable martensitic microstructure at the mouth temperature which enhance their flexibility [8], It is machined from a wire subjected to previously a thermo-mechanical process. The manufacturer has claimed that CM instrument has substantially enhanced flexibility and fatigue resistance which considerably decrease the incidence of instrument separation [9]. The Hyflex EDM (EDM) instruments (HEDM; Coltene/Whaledent AG, Altstätten, Switzerland) was processed using controlled electrical discharge machining method. The EDM process is a contact-free machining process used for manufacturing of parts that are difficult to machine with conventional grinding techniques [8, 10]. These techniques based on electrical sparks which provide a local melting to the materials with subsequent partial evaporation of minute parts of metals leaving superficial irregularities referred to “crater-like” surface finish. It is claimed that EDM instruments had high cyclic fatigue resistance [2, 8]. The ProTaper Next (PTN) instrument (PTN; Dentsply Maillefer, Ballaigues, Switzerland) is M-wire, thermally processed to enhance their flexibility and decrease the stresses concentration, its design include different tapers with a rectangular and off-centered cross-sectional area [11]. The Wave One Gold (WOG) instruments (WOG; Dentsply Maillefer, Ballaigues, Switzerland) used in reciprocating motion, is fabricated using M-wire using a heat-treatment process. It produces parallelogram-shaped cross-Sect. [12]. Recently introduced TruNatomy (TN) rotary instruments (TN; Dentsply Sirona, Baillagues, Switzerland) is subjected to heat treatment annealing, it provides a slender shape than improve the debridement process. Manufacture claim that TN instrument are extremely flexible and have a high cyclic fatigue resistance [13, 14]. Table 1 summarized the types of alloys, composition and properties of some Ni-Ti instruments [2, 6, 15].

Endodontic irrigants are used for a variety of biological, chemical, and mechanical purposes which are critical for successful root canal treatment [16–19]. It is advised to constantly perform root canal instrumentation under copious irrigating solution [19].When the irrigant encounters Ni-Ti instruments, this may lead instrument corrosion, deformation, surface roughness enhancing mechanical failure [5, 20, 21]. Amongst currently used irrigating solutions, NaOCl in 5.25% appears to the most commonly used and effective root canal irrigants [16, 22, 23].

The contact between Ni-Ti instruments and irrigants may be induce an instrumental corrosion and flaws accelerating instrument’s cyclic fatigue and failure [24].There is a strong relation between fracture mechanism and surface characteristics of rotary Ni-Ti instruments [25, 26].Repeated irrigation is reflected by reduction in Ni-Ti rotary endodontic instruments cutting efficiency and increase in their surface roughness [27, 28]. The surface roughness investigation of Ni-Ti rotary instruments offers convenient evidence concerning surface defects and performance [5]. AFM is one of the most widely used imaging tools used to assess surface topography accurately. AFM allows a topographic mapping of the surface using a non‑destructive probes [5, 29].

**Table 1:** Type of alloy and composition of Ni-Ti instruments

Ni-Ti system	Type of alloys	Composition
Hyflex CM	CM wire	Martensite with various amounts of austenite and R-phase
Hyflex EDM	CM wire using electrical discharge machining	No austenite phase
Protaper Next	M-Wire	Mainly austeNi-Tic phase with little amounts of R-phase and martensite
Wave One Gold	M-Wire using gold thermo-mechanical heat treatment	Mainly stable martensite or R-phase
TrueNatumy	Proprietary novel heat treatment technique	R-phase and martensitic transformation

Recently, there have been limited studies considering the surface roughness of the recently introduced Ni-Ti rotary instrument for assessment of this value before and after exposure to root canal irrigation [26, 30, 31]. Hence, the aim of the study is to evaluate the impact of endodontic irrigants on the surface roughness of several Ni-Ti rotary endodontic instruments. The null hypothesis was that there was no significant difference regarding surface roughness between Protaper Next (PTN), Hyflex CM (CM), Hyflex EDM (EDM), WaveOne Gold (WOG), and Trigonometry (TN) rotary Ni-Ti instruments before and after application of 5.25% sodium hypochlorite (NaOCl) irrigant solution.

## Methods

Sample size was calculated using G*Power (version 3.1.9.7) sample size calculator via means and standard deviations [32]. The estimated sample size required 10 endodontic rotary instruments in each group. The Medical Research Ethical Committee (MREC) of National Research Centre (NRC); Cairo, Egypt approved the research (Reference number: 24,312,012,023).

In the present study Protaper next (PTN; Dentsply Maillefer, Ballaigues, Switzerland), Hyflex CM; Coltene/Whaledent AG, Altstätten, Switzerland), Hyflex EDM; Coltene/Whaledent AG, Altstätten, Switzerland), WaveOne gold (WOG; Dentsply Maillefer, Ballaigues, Switzerland) and trunatomy (TN; Dentsply Maillefer, Ballaigues, Switzerland) were included. A new endodontic rotary instrument of each brand was examined and inspected under a scanning electron microscope (SEM) (Olympus soft imaging solutions, GMBH, Muenster, Germany) before performing the test, any distorted or faulty endodontic rotary instruments were excluded. The selected size of endodontic rotary instruments was 25 at the tip, with taper of 0.06 and a length of 25 mm. The irrigants employed were 5.25% NaOCl (Tianshi Biological Technology Co. Ltd, Henan, China).

Ten new endodontic rotary instruments of each brand were analyzed using AFM (Tosca 200 AFM, Anton Paar GmbH, Ostfildern, Germany). Analysis perormed using software Mountains 8 (Digital Surf, Besançon, France). AFM images were taken in tapping mode under ambient conditions at resolution 400, with rate 1 line/ second at angle 0 degree.

Analysis of each brand was performed before irrigation and then again after irrigation. Irrigation was done by immersion of each rotary instrument into 5.25% NaOCl for 15 min at 37 °C.

The dynamic immersion was done by rotating the endodontic rotary instruments attached to an endodontic motor (X-Smart Plus Endomotor, Dentsply Sirona, Ballaigues, Switzerland) and rotating freely at a constant speed (400 rpm) under constant torque (2.5 Ncm) into Eppendorf plastic tubes of 2 mL (Deltalab, S.L., Barcelona, Spain) containing 2 mL of 5.25% NaOCl irrigant solution for 15 min, which represent the clinical conditions during root canal therapy [33]. All the endodontic rotary instruments after removing from irrigant solution were rinsed with distilled water to neutralize the effect of irrigation and dried.

Each brand of endodontic rotary instrument was placed on the specimen stage of the AFM device, with the handle always in the same position. The same selected areas were examined before and after exposure to irrigants.

The endodontic rotary instruments were attached to a glass plate using a double-sided adhesive for evaluation of the cutting blade and the flute between the instruments. Each specimen was mounted on the AFM, and then 10 consecutive points positioned on a 4 mm section of the tip of each rotary instrument were examined; the scanned areas were 1 × 1 µm^2^ squares [34, 35]. The AFM images were recorded in tapping mode under ambient conditions. AFM analysis software was employed to process a three-dimensional (3D) image, and surface roughness parameters were evaluated using the Sa parameter.

The null hypothesis was that there would be no significant difference in mean Sa values among PTN, CM, EDM, WOG, and TN Ni-Ti instruments before and after immersion in 5.25% NaOCl irrigants.

### Statistical analysis

Data were analyzed using the Statistical Program for the Social Sciences (SPSS Inc., Chicago, IL, USA). Shapiro-Wilkes test was applied for the assessment of normality. Paired t-test was performed to detect the effect of the irrigant on the surface roughness of the endodontic instruments, by measuring the average surface roughness (Sa) before and after irrigation. While One-way ANOVA and Tukey tests were used to compare the surface roughness of the different instruments before irrigation. Similarly, the surface roughness of the different instruments was compared after irrigation. Significant difference was considered when P ≤ 0.05.

## Results

**Fig. 1:**
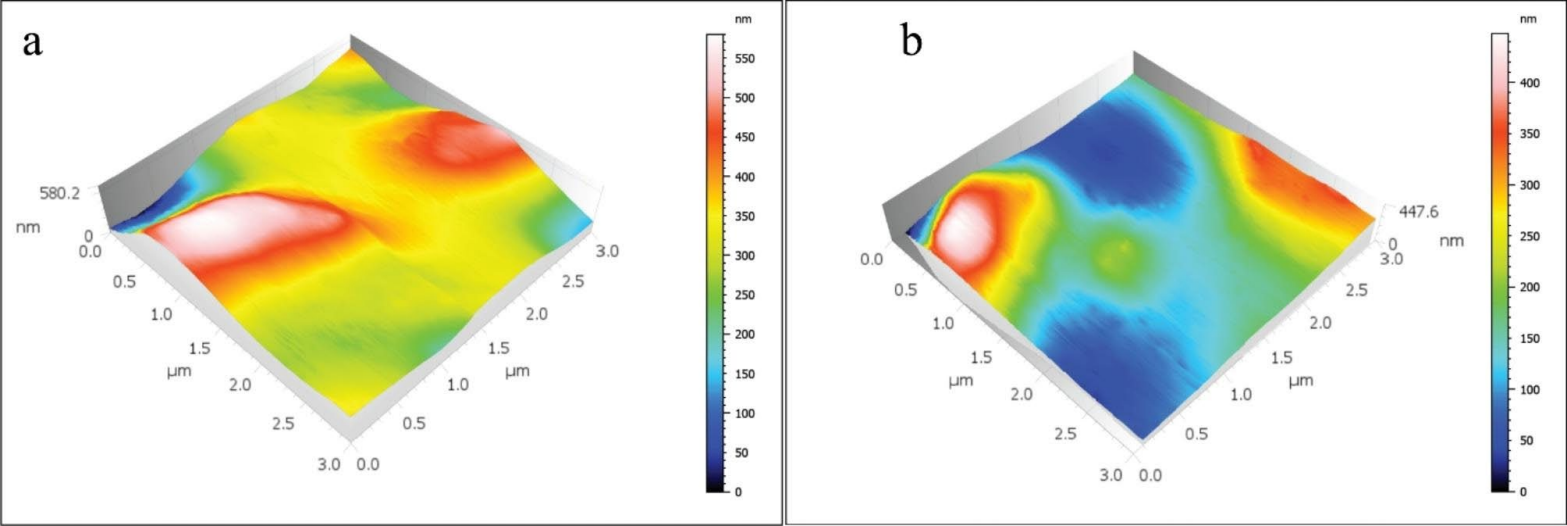
AFM imaging of CM instruments (**a**) before immersion in irrigants, (**b**) after immersion in irrigants

**Fig. 2:**
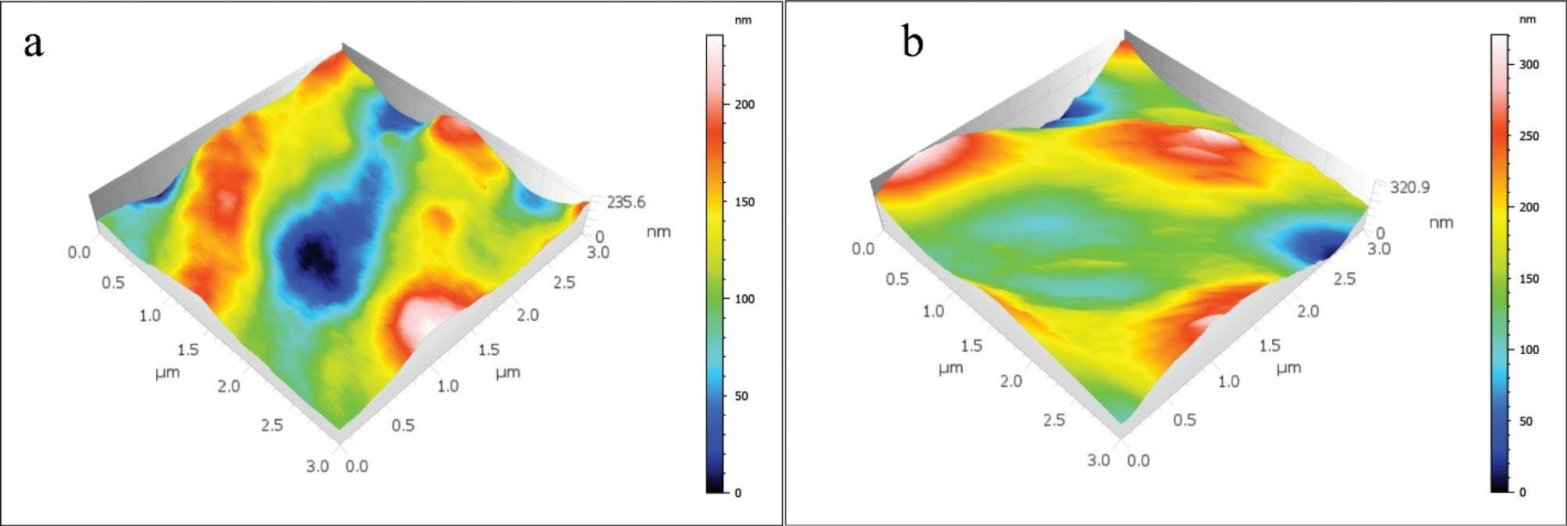
AFM imaging of EDM instruments (**a**) before immersion in irrigants, (**b**) after immersion in irrigants

**Fig. 3:**
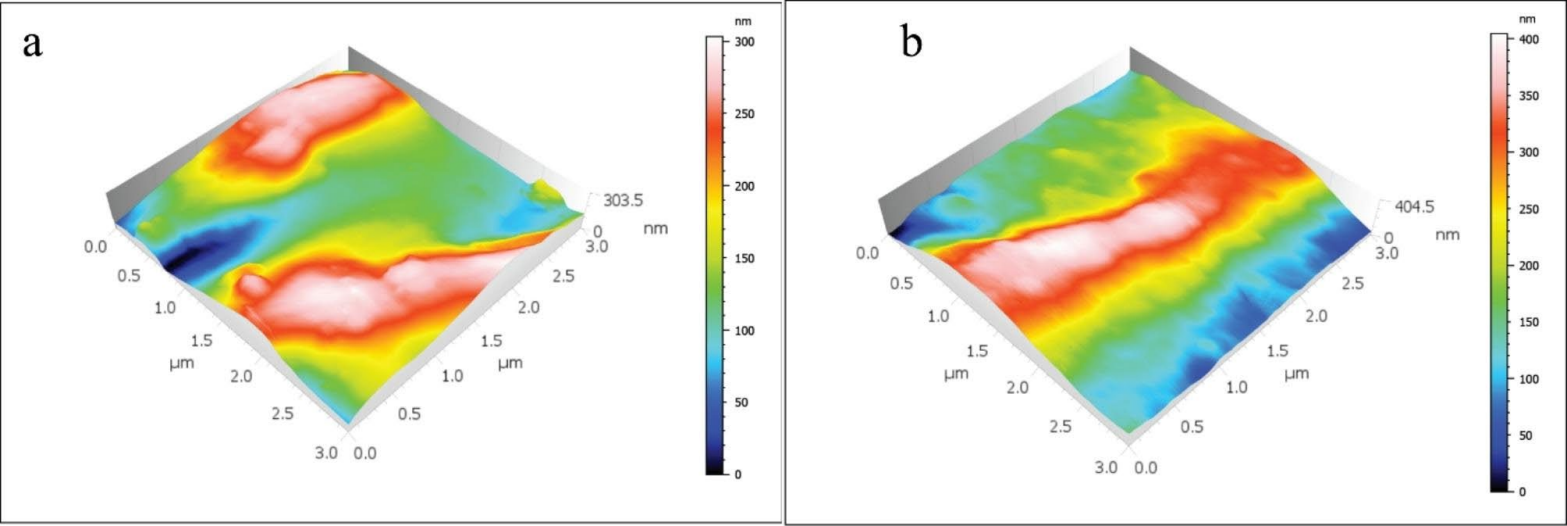
AFM imaging of PTN instruments (**a**) before immersion in irrigants, (**b**) after immersion in irrigants

**Fig. 4:**
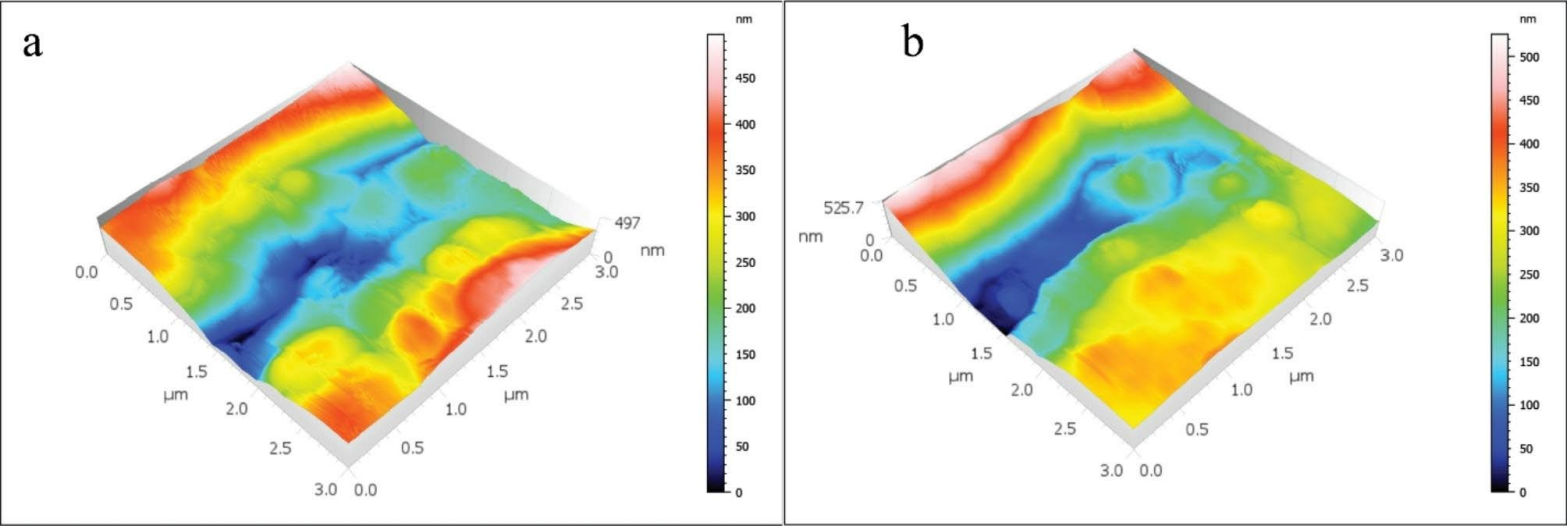
AFM imaging of WOG instruments (**a**) before immersion in irrigants, (**b**) after immersion in irrigants

**Fig. 5:**
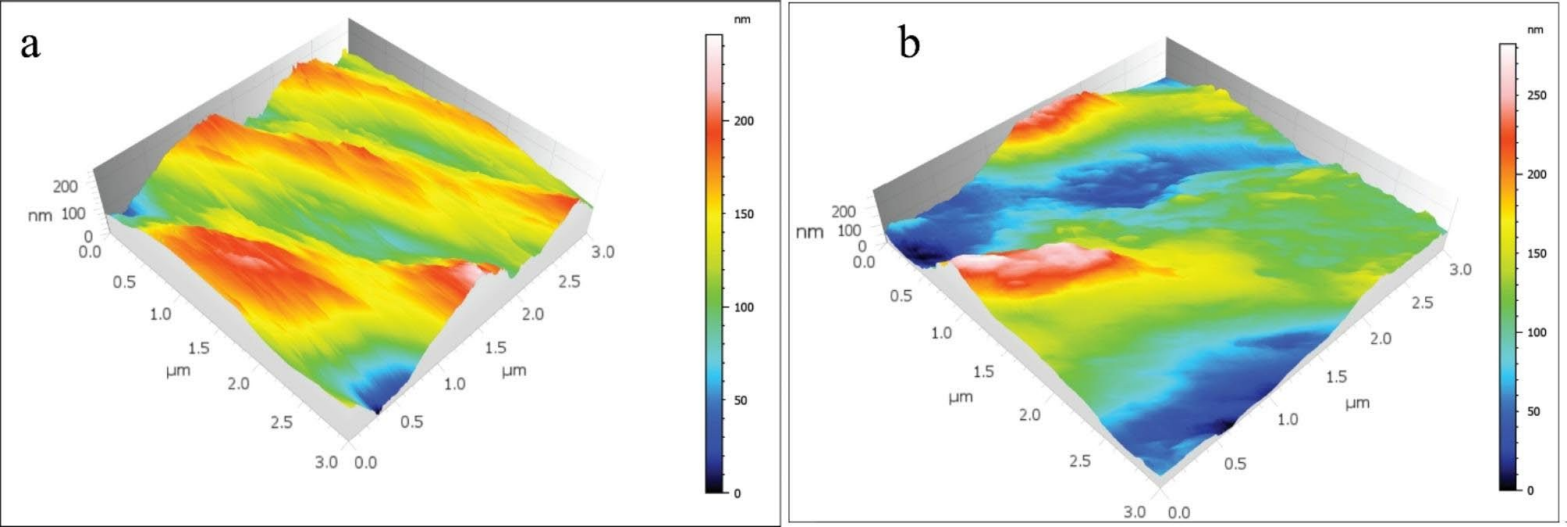
AFM imaging of TN instruments (**a**) before immersion in irrigants, (**b**) after immersion in irrigants

The mean and standard deviations of the Sa values are displayed in Table 2; Figs. 1, 2, 3, 4 and 5. There was a significant increase in surface roughness (Sa) in all tested rotary endodontic instruments after immersion in the irrigants.

Comparing the surface roughness of the different instruments before irrigant application amongst all groups showed that TN and PTN showed the least roughness with a P value = 0.0001* compared with other instruments with no significant difference between them (P = 0.7) while they were significantly different from the other instruments (P = 0.0001*). This is followed by WOG and CM with no significant difference between them (P = 1) while they were significantly different than the other instruments (P = 0.0001*). The EDM showed highest roughness compared to other instruments (P = 0.04 with CM and P = 0.0001* with the rest of instruments).

Comparing the surface roughness of the different instruments after irrigants application amongst all groups showed that CM and PTN showed the least roughness with a P value of 0.0001* compared with other instruments, with no significant difference between them (P = 1), while they were significantly different from the other instruments (P ≤ 0.01*). This is followed by EDM and TN with no significant difference between them (P = 0.8), while they were significantly different from the other instruments (P ≤ 0.04*). The WOG showed highest roughness compared to other instruments (P ≤ 0.03*) except TN; as no significant difference was detected among WOG and TN (P = 0.3).

**Table 2:** Mean and Standard Deviations of Sa parameter surface roughness before and after immersion of investigated rotary endodontic instruments (nm)

Rotary endodontic instruments	Surface roughness Sa values (nm)		P value
	Before immersion	After immersion	
CM	58.5^b^ ± 7	74.9^a^ ± 1.1	P = 0.04*
EDM	67.9^c^ ± 5	81.1^b^ ± 3.1	P = 0.001*
PTN	49.8^a^ ± 7.1	68.0^a^ ± 8	P = 0.0001*
WOG	66.6^b^ ± 8.6	84.1^c^ ± 2.4	P = 0.0001*
TN	38.8^a^ ± 1.3	83.1^bc^ ± 1.8	P = 0.0001*
P value	P = 0.0001*	P ≤ 0.04*	

Means in same row have no letters as they are only two groups (before and after irrigation), while means in same column with different letters indicate significance difference. *Corresponds to significant difference (P ≤ 0.05)

## Discussion

The current study is the first to evaluate the surface roughness of CM, EDM, PTN, WOG, and TN Ni-Ti instruments at the nanoscale before and after immersion in a 5.25% NaOCl irrigant solution using 3D AFM. Application of NaOCl at 5.25% is currently considered the gold standard for root canal irrigants [16, 23]. Surface features of Ni-Ti endodontic rotary instruments should be properly considered when evaluating their quality. Surface irregularities can potentially compromise corrosion resistance, and the cutting effectiveness of endodontic rotary instruments affects corrosion resistance [33, 36]. Ni-Ti endodontic rotary instruments corrosion starts with the selective removal of nickel from the alloy surface when the instrument is subjected to sodium NaOCl during the root canal treatment [14].

The surface irregularities could be assessed by evaluating the surface roughness [35].The surface roughness characterization of Ni-Ti endodontic rotary instruments delivers beneficial data concerning surface defects and performance [37]. Therefore, studies have been carried out using various techniques, including SEM, AFM and non-contact optical profilometry to examine the surface topography of endodontic instruments [5]. The SEM modality provided two-dimensional (2D) images were difficult to be administer for quantitative surface data [38]. AFM creates 3D topographical images that provide information about surface morphology and defects [39]. The AFM was a valuable, practical, and non-destructive technique for quantitative assessment of the surface topography of the Ni-Ti rotary instruments [30, 35].

Surface roughness might be expressed by various parameters. The modalities Sa, Sq, Sz were mostly to be utilized for endodontic instrumental surface analysis [40, 41]. The surface roughness average (Sa) topographical parameter represents the arithmetical mean height of the surface and expresses a class of amplitude parameters counting the properties of technical surfaces [42]. This parameter is generally used to evaluate surface roughness; it is used to investigate the vertical surface topography of endodontic rotary instruments and perform the comparison [32, 35]. It is well recognized that defects in surface microstructure may cause areas of stress concentration and the creation of cracks, which sequentially weaken the structural integrity of the Ni-Ti instrument [43–45].

The results revealed that the unused new brands of Ni-Ti instruments showed various degrees of Sa before irrigation. This observation may be due to the presence of surface defects and irregularities created during the process of manufacturing Ni-Ti instruments, which produce nano-scale surface irregularities, even in unused instruments [32, 35, 36, 46].

TN and PTN demonstrated the lowest Sa values. The lower Sa values of TN instruments may be attributed to their surface, which minimizes the residual machining defects from the fabrication procedure [47]. In addition, the thermo-mechanical processing gained by the special heat treatment of Ni-Ti wire could overcome the machining procedure faults through modification of their crystalline phase [2, 48]. Moreover, the lower Sa values of PTN instruments may be due to their fabrication from M-wire, which is thermally treated during manufacturing to improve flexibility and increase fatigue resistance [5, 49].

WOG and CM demonstrated higher Sa values than other instruments. These findings may be due to the fact that WOG manufacturing undergoes heat treatment of the alloy (gold wire) to machine a parallelogram-shaped cross-section, which may affect the surface characteristics of NiTi, leading to a rougher or even porous surface [5]. Similarly, the cause of the rougher surfaces of CM instruments may be attributed to their production procedure in a special thermo-mechanical process that aims to increase the flexibility of traditional NiTi files [50]. However, the highest Sa values of EDM may be due to the electrical discharge machining technology, which is based on the vaporizing and melting of the small particles on the surface by the action of electric sparks and shaping, which induces surface irregularities [50, 51].

Concerning Sa after irrigation, amongst all tested Ni-Ti instruments, all the tested Ni-Ti rotary endodontic instruments exhibited varying amounts of Sa values after exposure to irrigants. These findings may be due to the deterioration effect of NaOCl on the Ni-Ti instrument surface. The exposure of the metal to NaOCl irrigants may lead to corrosion and the subsequent production of cracks and pits that affect the surface integrity of the Ni-Ti instrument [1, 44, 52].

The results showed that CM and PTN demonstrated the lowest Sa values; this finding may be due to the fact that the CM instrument had a uniform surface topography [51]. These findings may also be due to the martensite grains of M-wire and their smaller grain size than other Ni-Ti wires, which are responsible for increasing their wear resistance and strength. Also, they concluded that the smaller grain sizes of the alloy can inhibit crack initiation through the grain boundaries [28, 53, 54].

Moreover, PTN instruments, which were produced from an M-wire and subjected to pre-machining heat treatment, may be the cause of reducing the surface cracks upon instrumentation [55]. Furthermore, the results showed that EDM and TN demonstrated a higher Sa value. The electrical sparks used during the fabrication process of EDM instruments may be responsible for the creation of surface roughness that could improve the cutting efficiency of tools but produce micropores, which are referred to as corrosion pits, which are associated with crack initiation and encourage corrosion by the action of NaOCl [15, 51, 53, 56].

While TN exhibited more surface roughness, which may be explained by the fact that proprietary post-machining heat treatment manufacturing processes may induce inherent characteristics that reduce their resistance to corrosion on exposure to irrigants [15, 57]. Our results showed that WOG demonstrated the highest SA value. This may be attributed to the fact that WOG undergoes thermal treatment before and after instrument machining [58]. Additionally, heat treatment employed during manufacturing may alter the Ni-Ti surface characteristics, resulting in a rough or porous surface [5, 31, 59].

In summary, it was noted that manufacturers use various methods of surface treatment, such as ion implantation, thermal nitridation, cryogenic treatment, nitride coating, and electropolishing, to tackle processing defects [57, 60]. But these processes may counteract the irrigation steps, interact with the chlorine ions in the irrigation solutions, and affect the surface integrity.

The present study has evaluated the endodontic rotary instruments in an in vitro situation, which is considered a limitation. Although operating endodontic rotary instruments in tooth canals was more appropriate for the clinical situation, the operation and immersion of endodontic rotary instruments were performed in a plastic tube to standardize the experimental comparative study. Moreover, the absence of friction between endodontic rotary instruments and dentin may lead to lower wear results, which may represent a limitation of this in vitro study. Further studies are recommended to be conducted using different concentrations and types of irrigants over different periods of immersion. Moreover, the changes in the chemistry of the Ni-Ti surface in response to exposure to irrigants may be useful to investigate using chemical elemental analysis.

## Conclusions

The new TN and PTN Ni-Ti instruments showed the least surface roughness. Irrigations with 5.25% NaOCl for 15 min. affect the surface roughness of all tested Ni-Ti rotary endodontic instruments. CM and PTN Ni-Ti instruments exhibited the least surface roughness after irrigant exposure.

## Data Availability

The data that support the findings of this study are available from the corresponding author upon reasonable request.

## Abbreviations

Ni-Ti: Nickel-titanium
PTN: Protaper next
CM: Hyflex CM
EDM: Hyflex EDM
WOG: WaveOne gold
TN: Trunatomy
NaOCl: Sodium hypochlorite
Sa: Surface roughness average
AFM: Atomic Force Microscope
MREC: The Medical Research Ethical Committee
NRC: National Research Centre
3D: Three-dimension
2D: Two-dimension

## References

[1] Alfawaz H, Alqedairi A, Alhamdan M, Alkhzim N, Alfarraj S, Jamleh A (2022). Effect of NaOCl and EDTA irrigating solutions on the cyclic fatigue resistance of EdgeTaper Platinum instruments. BMC Oral Health.

[2] Hamdy TM, Galal M, Ismail AG, Abdelraouf RM (2019). Evaluation of flexibility, microstructure and elemental analysis of some contemporary nickel-titanium rotary instruments. Open Access Maced J Med Sci.

[3] Radwański M, Łęski M, Pawlicka H (2018). The influence of the manufacturing process of rotary files on the shaping of L-shaped canals. Dent Med Probl.

[4] Parashos P, Messer HH (2006). Rotary NiTi instrument fracture and its consequences. J Endod.

[5] Zafar MS (2021). Impact of endodontic instrumentation on Surface Roughness of various Nickel-Titanium Rotary Files. Eur J Dent.

[6] Tabassum S, Zafar K, Umer F (2019). Nickel-titanium rotary file systems: what’s new?. Eur Endodontic J.

[7] R N (2018). NiTi Endodontics: contemporary views reviewed. Austin J Dent.

[8] Pirani C, Iacono F, Generali L, Sassatelli P, Nucci C, Lusvarghi L (2016). HyFlex EDM: superficial features, metallurgical analysis and fatigue resistance of innovative electro discharge machined NiTi rotary instruments. Int Endod J.

[9] Caicedo DR, Clark SJ (2016). Clinical HyFlex ® CM rotary files: an excellent innovation for endodontic treatment. Endod Prac.

[10] Singh H, Hyflex CM, Files EDM. Revolutionizing the art and science of endodontics. J Dent Heal Oral Disord Ther. 2016;5.

[11] Çelik G, Kisacik F, Yilmaz EF, Mersinlioǧlu A, Ertuǧrul IF, Orhan H. A comparative study of root canal shaping using protaper universal and protaper next rotary files in preclinical dental education. PeerJ. 2019;2019. 10.7717/peerj.7419 PMC6705381 31489262

[12] Bueno CSP, Oliveira DP, Pelegrine RA, Fontana CE, Rocha DGP, Gutmann JL (2020). Fracture incidence of WaveOne Gold files: a prospective clinical study. Int Endod J.

[13] Reddy BN, Murugesan S, Basheer SN, Kumar R, Kumar V, Selvaraj S (2021). Comparison of cyclic fatigue resistance of Novel TruNatomy files with conventional endodontic files: an in Vitro SEM study. J Contemp Dent Pract.

[14] Ba-Hattab R, Almohareb RA, Alkhalaf R, Binnjefan S, Sulayem M, Barakat RM. The Impact of Multiple Autoclave Cycles on the Surface Roughness of Thermally Treated Nickel-Titanium Endodontic Files. Adv Mater Sci Eng. 2022;2022.

[15] Chan WS, Gulati K, Peters OA (2023). Advancing Nitinol: from heat treatment to surface functionalization for nickel–titanium (NiTi) instruments in endodontics. Bioact Mater.

[16] Zehnder M (2006). Root Canal Irrigants. J Endod.

[17] Hamdy TM, Abdelnabi A, Othman MS, Bayoumi RE, Abdelraouf RM (2023). Effect of different mouthwashes on the Surface Microhardness and Color Stability of Dental Nanohybrid Resin Composite. Polym (Basel).

[18] Tashkandi N, Alghamdi F (2022). Effect of Chemical Debridement and Irrigant activation on Endodontic Treatment Outcomes: an updated overview. Cureus.

[19] Haapasalo M, Shen Y, Qian W, Gao Y (2010). Irrigation in endodontics. Dental Clin N Am.

[20] Peters OA, Roehlike JO, Baumann MA (2007). Effect of immersion in Sodium Hypochlorite on torque and fatigue resistance of Nickel-Titanium Instruments. J Endod.

[21] Bonaccorso A, Tripi TR, Rondelli G, Condorelli GG, Cantatore G, Schäfer E (2008). Pitting Corrosion Resistance of Nickel-Titanium Rotary Instruments with different surface treatments in 17% ethylenediaminetetraacetic acid and Sodium Chloride Solutions. J Endod.

[22] Omar N, Abdelraouf RM, Hamdy TM (2023). Effect of different root canal irrigants on push- out bond strength of two novel root-end filling materials. BMC Oral Health.

[23] Palazzi F, Morra M, Mohammadi Z, Grandini S, Giardino L (2012). Comparison of the surface tension of 5.25% sodium hypochlorite solution with three new sodium hypochlorite-based endodontic irrigants. Int Endod J.

[24] Amato M, Pantaleo G, Abdellatif D, Blasi A, Lo Giudice R, Iandolo A (2017). Evaluation of cyclic fatigue resistance of modern Nickel-Titanium rotary instruments with continuous rotation. G Ital Endod.

[25] Valois CRA, Silva LP, Azevedo RB (2008). Multiple autoclave cycles affect the Surface of Rotary Nickel-Titanium Files: an Atomic Force Microscopy Study. J Endod.

[26] Van Pham K, Vo CQ. A new method for assessment of nickel-titanium endodontic instrument surface roughness using field emission scanning electronic microscope. BMC Oral Health. 2020;20. 10.1186/s12903-020-01233-0 PMC7461342 32867760

[27] Arslan H, Doğanay Yıldız E, Taş G, Karataş E, Tepecik E (2020). Effects of continuous irrigation at room temperature or + 4^o^C on the cyclic fatigue resistance of K3XF instruments. J Dent Res Dent Clin Dent Prospects.

[28] Yılmaz K, Uslu G, Özyürek T (2018). Effect of multiple autoclave cycles on the surface roughness of HyFlex CM and HyFlex EDM files: an atomic force microscopy study. Clin Oral Investig.

[29] Tsenova-Ilieva I, Simeonova S, Karova E (2022). Atomic force microscopy study on the effect of different irrigation regimens on the surface roughness of human root canal dentin. Niger J Clin Pract.

[30] Inan U, Aydin C, Uzun O, Topuz O, Alacam T (2007). Evaluation of the surface characteristics of used and new ProTaper Instruments: an Atomic Force Microscopy Study. J Endod.

[31] Özyürek T, Yılmaz K, Uslu G, Plotino G. The effect of root canal preparation on the surface roughness of WaveOne and WaveOne Gold files: atomic force microscopy study. Restor Dent Endod. 2018;43. 10.5395/rde.2018.43.e10 PMC5816987 29487840

[32] Cai JJ, Tang XN, Ge JY (2017). Effect of irrigation on surface roughness and fatigue resistance of controlled memory wire nickel-titanium instruments. Int Endod J.

[33] Han-Hsing Lin J, Karabucak B, Lee SM (2021). Effect of sodium hypochlorite on conventional and heat-treated nickel-titanium endodontic rotary instruments – an in vitro study. J Dent Sci.

[34] Donnermeyer D, Vahdat-Pajouh N, Schäfer E, Dammaschke T (2019). Influence of the final irrigation solution on the push-out bond strength of calcium silicate-based, epoxy resin-based and silicone-based endodontic sealers. Odontology.

[35] Fatma Y, Ozgur U (2014). Evaluation of surface topography changes in three NiTi file systems using rotary and reciprocal motion: an atomic force microscopy study. Microsc Res Tech.

[36] Stošić N, Popović J, Apostolović MA, Mitić A, Nikolić M, Barac R (2021). Ultrastructural analysis of the surface changes on the nickel-titanium endodontic instruments after multiple use. Acta Fac Medicae Naissensis.

[37] Jamaluddin R, Lih TC, Mansor AF, Azmi AI, Hamidon R. Surface roughness analysis of NiTi alloy in electrical discharge coating process. In: IOP Conference Series: Materials Science and Engineering. 2020.

[38] Sağlam BC, Görgül G (2015). Evaluation of surface alterations in different retreatment nickel-titanium files: AFM and SEM study. Microsc Res Tech.

[39] Asylum R. Measuring surface roughness with Atomic Force Microscopy. Asylum Res an Oxford Instruments Co. 2013;:2–3.

[40] Van Pham K, Vo CQ (2020). A new method for assessment of nickel-titanium endodontic instrument surface roughness using field emission scanning electronic microscope. BMC Oral Health.

[41] Lopes HP, Elias CN, Vieira MVB, Vieira VTL, De Souza LC, Dos Santos AL (2016). Influence of Surface Roughness on the fatigue life of Nickel-Titanium Rotary Endodontic Instruments. J Endod.

[42] Löberg J, Mattisson I, Hansson S, Ahlberg E (2010). Characterisation of Titanium Dental Implants I: critical Assessment of Surface Roughness Parameters. Open Biomater J.

[43] Sonntag D, Peters OA (2007). Effect of Prion Decontamination Protocols on Nickel-Titanium Rotary Surfaces. J Endod.

[44] Berutti E, Angelini E, Rigolone M, Migliaretti G, Pasqualini D (2006). Influence of sodium hypochlorite on fracture properties and corrosion of ProTaper Rotary instruments. Int Endod J.

[45] Paul S, Anto T, Anil A, KM C (2018). Evaluation of the Effect of various concentration of Sodium Hypochlorite on the Surface Roughness of ProTaper Rotary Files using Atomic Force Microscopy: an in Vitro Study. Conserv Dent Endod J.

[46] Alexandrou GB, Chrissafis K, Vasiliadis LP, Pavlidou E, Polychroniadis EK (2006). SEM observations and Differential scanning calorimetric studies of New and Sterilized Nickel-Titanium Rotary Endodontic Instruments. J Endod.

[47] Arantes WB, Da Silva CM, Lage-Marques JL, Habitante S, Da Rosa LCL, De Medeiros JMF (2014). SEM analysis of defects and wear on Ni-Ti rotary instruments. Scanning.

[48] Elnaghy AM, Elsaka SE, Mandorah AO (2020). In vitro comparison of cyclic fatigue resistance of TruNatomy in single and double curvature canals compared with different nickel-titanium rotary instruments. BMC Oral Health.

[49] Gao Y, Gutmann JL, Wilkinson K, Maxwell R, Ammon D (2012). Evaluation of the impact of raw materials on the fatigue and mechanical properties of profile vortex rotary instruments. J Endod.

[50] Uslu G, Özyürek T, Yılmaz K (2018). Comparison of alterations in the Surface Topographies of HyFlex CM and HyFlex EDM nickel-titanium files after Root Canal Preparation: A three-dimensional Optical Profilometry Study. J Endod.

[51] Iacono F, Pirani C, Generali L, Sassatelli P, Nucci C, Gandolfi MG (2016). Wear analysis and cyclic fatigue resistance of electro discharge machined NiTi rotary instruments. G Ital Endod.

[52] Huang X, Shen Y, Wei X, Haapasalo M (2017). Fatigue resistance of nickel-titanium Instruments exposed to high-concentration Hypochlorite. J Endod.

[53] Bennett J, Chung KH, Fong H, Johnson J, Paranjpe A (2017). Analysis of surface characteristics of protaper universal and protaper next instruments by scanning electron microscopy. J Clin Exp Dent.

[54] Ye J, Gao Y (2012). Metallurgical characterization of M-Wire nickel-titanium shape memory alloy used for endodontic rotary instruments during low-cycle fatigue. J Endod.

[55] Elemam R, Capelas J, Vieira M (2016). Effect of repeated use on Topographical features of ProTaper Next Endodontic Rotary file. J Int Oral Heal.

[56] Mahajan A, Singh G, Devgan S, Sidhu SS (2021). EDM performance characteristics and electrochemical corrosion analysis of co-cr alloy and duplex stainless steel: a comparative study. Proc Inst Mech Eng Part E J Process Mech Eng.

[57] Jose J, Khandelwal A, Siddique R (2021). Qualitative Assessment of the Surface Topographic Changes of XP-endo shaper and TruNatomy files after exposure to Sodium Hypochlorite and Ethylenediaminetetraacetic Acid. Eur Endod J.

[58] Srivastava S. Current strategies in metallurgical advances of Rotary NiTi Instruments: a review. J Dent Heal Oral Disord Ther. 2018;9.

[59] Firstov GS, Vitchev RG, Kumar H, Blanpain B, Van Humbeeck J (2002). Surface oxidation of NiTi shape memory alloy. Biomaterials.

[60] Mohammadi Z, Soltani MK, Shalavi S, Asgary S. A review of the various surface treatments of NiTi instruments. Iran Endodontic J. 2014. PMC4224758 25386201

